# An all-*trans*-retinal-binding opsin peropsin as a potential dark-active and light-inactivated G protein-coupled receptor

**DOI:** 10.1038/s41598-018-21946-1

**Published:** 2018-02-23

**Authors:** Takashi Nagata, Mitsumasa Koyanagi, Robert Lucas, Akihisa Terakita

**Affiliations:** 1 Department of Biology and Geosciences, Graduate School of Science, Osaka City University, 3-3-138 Sugimoto, Sumiyoshi-ku, Osaka, 558–8585 Japan; 2 The OCU Advanced Research Institute for Natural Science and Technology (OCARINA), Osaka City University, 3-3-138 Sugimoto, Sumiyoshi-ku, Osaka, 558–8585 Japan; 3 Faculty of Biology, Medicine and Health, The University of Manchester, Manchester, M13 9PT UK

## Abstract

Peropsin or retinal pigment epithelium-derived rhodopsin homolog, found in many animals, belongs to the opsin family. Most opsins bind to 11-*cis*-retinal as a chromophore and act as light-activated G protein-coupled receptors. Some peropsins, however, bind all-*trans*-retinal and isomerise it into 11-*cis* form by light, and peropsin has been suggested to supply other visual opsins with 11-*cis*-retinal. Additionally, peropsin has some amino acid sequence motifs that are highly conserved among G protein-coupled opsins. Here, using chimeric mutant peropsins, we found that peropsin potentially generates an “active form” that drives G-protein signalling in the dark by binding to all-*trans*-retinal and that the active form photo-converts to an inactive form containing 11-*cis*-retinal. Comparative spectroscopic analysis demonstrated that spider peropsin exhibited catalytic efficiency for retinal photoisomerisation that was much lower than a retinal photoisomerase, squid retinochrome. The chimeric peropsins, constructed by replacing the third intracellular loop region with that of Gs- or Gi-coupled opsin, were active and drove Gs- or Gi-mediated signalling in the dark, respectively, and were inactivated upon illumination in mammalian cultured cells. These results suggest that peropsin acts as a dark-active, light-inactivated G protein-coupled receptor and is useful as a novel optogenetic tool.

## Introduction

Rhodopsin and related photopigments consist of a protein moiety, opsin, and chromophore retinal and serve as light-sensing proteins typically found in the eyes of many animals. Thousands of opsin genes have been identified and are phylogenetically classified into eight groups^1,2^. Opsins belonging to six groups are known to serve as light-sensing G protein-coupled receptors (GPCRs) coupled to one or more of heterotrimeric G protein subtypes including transducin (Gt), Go, Gi, Gq, and Gs. These opsins, with few exceptions, bind to an 11-*cis* form as a chromophore retinal to form opsin-based pigments. In the 11-*cis*-retinal-binding forms of these pigments, known as dark states, the chromophore isomerises into all-*trans* form upon absorption of light, which triggers conformational changes in opsins and leads to the formation of photoproducts, the forms that activate G proteins^1,2^. In contrast, members of the retinochrome and RGR group bind to all-*trans*-retinal in the dark and isomerises the retinal into the 11-*cis* form with light^3,4^. Several lines of evidence suggest that retinochrome in molluscan retinas photoisomerises all-*trans*-retinal to the 11-*cis* form, which is used to form visual pigments^4–6^. RGR is suggested to be involved in light-dependent recovery of 11-*cis*-retinal in the mammalian retinal pigment epithelium by mediating translocation of all-*trans*-retinyl esters^7,8^. However, there is no direct evidence showing that retinochrome or RGR can activate G proteins.

Unlike the seven groups described above, little is known about the functions of peropsin group proteins. Peropsin, or retinal pigment epithelium-derived rhodopsin homolog, was first identified in the mouse retinal pigment epithelium^9^ and is found in nearly all vertebrate classes^10^. We previously identified peropsin genes in an amphioxus, *Branchiostoma belcheri*, and a jumping spider, *Hasarius adansoni*, revealing that invertebrates also possess peropsin genes^11,12^. Amphioxus and spider peropsins bind to all-*trans*-retinal as a chromophore and isomerise it into 11-*cis* form upon illumination^11,12^. This all-*trans*-to-11-*cis* photoisomerisation suggests that peropsin functions as a retinal photoisomerase similar to retinochrome. Additionally, we revealed that the photoproduct (i.e. 11-*cis*-retinal-binding form) of spider peropsin is thermally stable and does not release the chromophore retinal^12^. In addition, peropsins of many animals contain DRY and NPxxY motifs, which are amino acid sequences that are highly conserved among GPCRs and involved in the activation of G proteins^12^. These characteristics are common among opsins that activate G proteins and therefore suggest that peropsin functions as a light-sensing GPCR.

In this study, we examined the function of peropsin by characterising its molecular properties. To examine whether peropsin acts as a retinal photoisomerase, similar to retinochrome, we compared the catalytic efficiency for enzymatic retinal photoisomerisation between spider peropsin and squid retinochrome and found that spider peropsin does not catalyse retinal isomerisation as efficiently as retinochrome. Although we failed to observe any considerable changes in second messenger levels regulated by Gq, Gs, or Gi signalling in cultured cells expressing spider peropsin, some peropsin mutants in which the intracellular regions were replaced with those of G protein-coupled opsins exhibited activation of G proteins, suggesting that peropsin activates G proteins.

## Results

### Low catalytic activity of peropsin for all-*trans*-to-11-*cis* isomerisation of retinal

**Figure 1:**
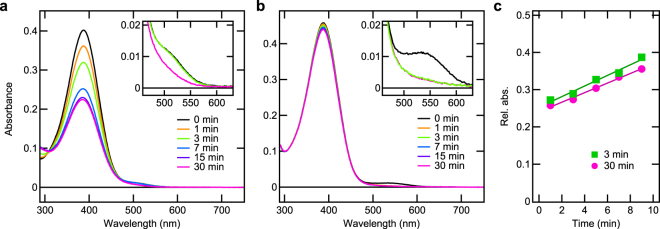
Photoisomerisation of all-*trans*-retinal by squid retinochrome and spider peropsin. (**a**,**b**) Absorption spectra of mixtures of all-*trans*-retinal and squid retinochrome (**a**) or spider peropsin (**b**) before and after illumination for 1, 3, 7, 15, and 30 min. Insets: absorption spectra around λmax of retinochrome (≈500 nm) and peropsin (≈540 nm). Note that absorbance of the dark state of retinochrome showed no decrease after illumination for 3 min because it completely regenerated with all-*trans*-retinal by the time of the measurement after illumination, namely within ≈1 min. (**c**) Regeneration processes of the dark state of spider peropsin during dark incubation after illumination for 3 and 30 min. Relative values of absorbance around 540 nm, normalized to that before illumination, are plotted against the time of dark incubation and fitted with linear functions.

We first compared the catalytic activities for photoisomerisation of all-*trans*-retinal as a substrate to the 11-*cis* form between spider peropsin and squid retinochrome. A previous study^13^ showed that squid retinochrome isomerases excess all-*trans-*retinal into 11-*cis* form upon illumination, resulting in a decrease in absorption of retinal with a slight blue shift. Approximately 0.01 A.U. (i.e. absorbance unit) or 160 nM of squid retinochrome and 0.4 A.U. or 22 μM of all-*trans*-retinal were mixed and illuminated with yellow light (>510 nm; Fig. 1a). The absorbance around 390 nm decreased by approximately 40% with a slight blue shift of the peak after illumination for 15 min, in agreement with a previous study^13^, demonstrating that all-*trans*-to-11-*cis* isomerisation of retinal was catalysed by retinochrome. Notably, illumination of all-*trans*-retinal without retinochrome for 30 min only decreased the absorbance around 390 nm by approximately 1.5% (Supplementary Fig. S1). In the presence of spider peropsin under experimental conditions similar to those for retinochrome, the illumination with orange light (>550 nm) caused the absorbance around 390 nm to decrease by approximately 4%, demonstrating that only a small amount of all-*trans*-retinal was isomerised (Fig. 1b). The regeneration rates of the dark state of spider peropsin (λmax ≈540 nm) after illumination for 3 and 30 min were similar (Fig. 1c; Supplementary Fig. S2), demonstrating that spider peropsin was not denatured during the 30-min illumination. Therefore, spider peropsin has a much lower catalytic activity for retinal photoisomerisation compared to squid retinochrome. Such low activity of spider peropsin can be explained by the slow regeneration rate. After 3-min illumination, nearly 100% (i.e. 0.01 A.U.) of the dark state of retinochrome was regenerated within ≈1 min (inset, Fig. 1a), whereas only approximately 10% (i.e. 0.001 A.U.) of spider peropsin was regenerated in 8 min (Fig. 1c), giving a regeneration rate of approximately 1.3% per min, which indicates that regeneration of spider peropsin was much slower than that of squid retinochrome. It is possible that stable binding between 11-*cis*-chromophore and the protein in the photoproduct^12^ inhibited the replacement of 11-*cis* form with the all-*trans* form to regenerate the dark state spider peropsin.

### No detectable activation of major G protein subclasses by spider peropsin

**Figure 2:**
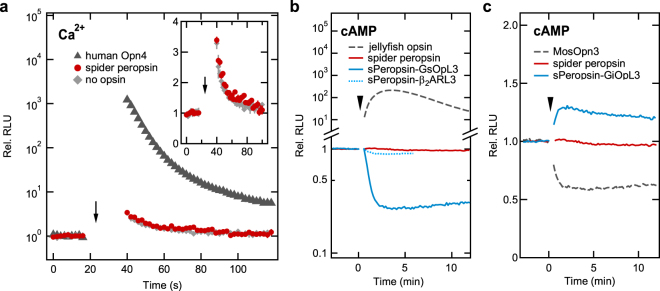
Bioluminescent assays for light-dependent changes in intracellular Ca^2+^ and cAMP levels. (**a**) Aequorin Ca^2+^ assay with HEK293 cells heterologously expressing human Opn4, spider peropsin, or no opsin. The inset shows the same data of spider peropsin and no opsin on a linear scale. Averaged values for three samples are shown with error bars (s.e.m.). (**b**) GloSensor cAMP assay with HEK293 cells expressing jellyfish opsin, spider peropsin, sPeropsin-GsOpL3, or sPeropsin-β_2_ARL3. (**c**) GloSensor cAMP assay with HEK293 cells expressing MosOpn3 or sPeropsin-GiOpL3. The same data of spider peropsin shown in Fig. 2b is shown here for comparison. Cells were illuminated with white light (**a**; arrows) or green light (**b**,**c**; arrowheads).

Low catalytic activity of spider peropsin for retinal photoisomerisation suggests an alternative possibility that peropsin functions as a light-sensing GPCR. First, we performed a bioluminescent Ca^2+^ assay using aequorin to investigate whether spider peropsin can drive Gq signalling in cultured cells. A Gq-coupled opsin, human Opn4, evoked a massive light-dependent increase in luminescence, which is consistent with a previous report^14^ and showed that activation of Gq increased intracellular Ca^2+^ concentrations (Fig. 2a). In contrast, we did not detect any considerable change in luminescence upon illumination with spider peropsin-expressing cells compared to cells expressing no opsin (Fig. 2a), demonstrating that activation of Gq by spider peropsin did not occur under this condition.

We next investigated whether peropsin activates Gs or Gi by conducting a GloSensor cAMP assay. Cells expressing Gs-coupled box jellyfish opsin^15^, which were preincubated with 11-*cis*-retinal overnight, exhibited a light-dependent increase in luminescence, showing a typical increase in intracellular cAMP levels through Gs signalling (Fig. 2b). Spider peropsin-expressing cells, which were preincubated with all-*trans*-retinal, exhibited no considerable changes in cAMP levels (Fig. 2b), indicating that wild-type spider peropsin does not couple to Gs or Gi under this condition. To further investigate the possibility that spider peropsin activates G proteins, we introduced a mutation to enhance the activation ability of Gs into spider peropsin, according to our previous finding that replacement of the third intracellular loop region (IL3) with the corresponding region of the Gs-coupled jellyfish opsin enables various opsins to activate Gs in a light-dependent manner^16^. Interestingly, cells expressing the chimeric spider peropsin mutant in which IL3 was replaced with that of the jellyfish opsin (referred to as sPeropsin-GsOpL3; see Supplementary Fig. S3 for the amino acid sequence), preincubated with all-*trans*-retinal, responded to light, in contrast to wild-type peropsin-expressing cells (Fig. 2b). Surprisingly, light evoked a decrease in cAMP levels in cells expressing sPeropsin-GsOpL3 in contrast to in jellyfish opsin-expressing cells (Fig. 2b). The most plausible explanation for this result is that the dark state of sPeropsin-GsOpL3 may activate Gs, while its photoproduct does not.

To examine this observation, we generated another spider peropsin mutant in which IL3 was replaced with the sequence of β_2_ adrenergic receptor (sPeropsin-β_2_ARL3; Supplementary Fig. S3), which is known as a prototypical Gs-coupled GPCR, because a previous study showed that a bovine rhodopsin mutant in which the IL3 was replaced with that of β_2_ adrenergic receptor evoked a light-dependent increase in cAMP levels in HEK293 cells^17^. Cells expressing sPeropsin-β_2_ARL3 exhibited a smaller but clear light-dependent decrease in cAMP levels, which is similar to the result of sPeropsin-GsOpL3 (Fig. 2b, Supplementary Fig. S4). We next investigated whether a peropsin mutant containing the IL3 of a Gi-coupled opsin induced an opposite light-dependent change in cAMP levels compared to the Gi-coupled opsin. Illumination of cells expressing a mosquito Gi/o-coupled opsin, MosOpn3, caused a light-dependent decrease in cAMP, presumably via Gi signalling (Fig. 2c), as previously shown^18^. As expected, the spider peropsin mutant with MosOpn3 IL3 (sPeropsin-GiOpL3; Supplementary Fig. S3) evoked a light-dependent rise in cAMP levels, as opposed to MosOpn3 (Fig. 2c). Taken together, these results suggest that the dark states of spider peropsin mutants activated Gs or Gi and these activation abilities were at least partially lost in their photoproducts, indicating that the mutants served as dark-active, light-inactivated GPCRs.

### Further investigation of chimeric peropsin mutants coupled to G protein in a dark-active and light-inactivated manner

**Figure 3:**
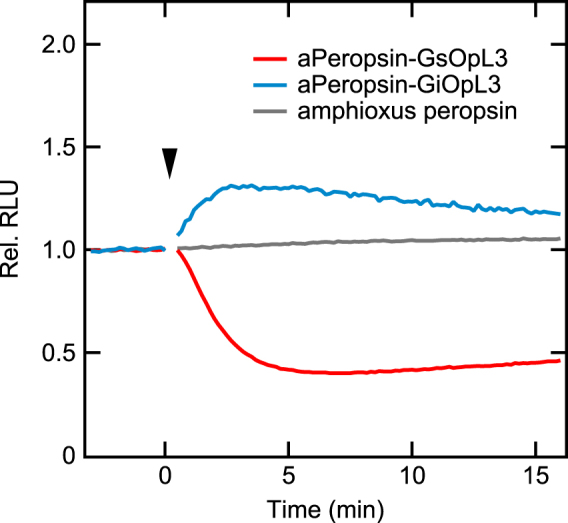
Light-dependent changes in cAMP level in cells expressing amphioxus peropsin and its mutants. GloSensor cAMP assays with cells expressing amphioxus peropsin, aPeropsin-GsOpL3, or aPeropsin-GiOpL3. Cells were illuminated with green light (arrowhead).

Based on the above results for spider peropsin, we next generated mutant proteins based on amphioxus peropsin containing IL3 of the jellyfish opsin (aPeropsin-GsOpL3) or MosOpn3 (aPeropsin-GiOpL3; Supplementary Fig. S3) to investigate whether peropsin of a deuterostome also produces similar results. aPeropsin-GsOpL3 and aPeropsin-GiOpL3 evoked a light-dependent decrease and increase in cAMP levels, respectively, similar to sPeropsin-GsOpL3 and sPeropsin-GiOpL3 (Fig. 3), suggesting that the unique molecular feature that enables replacement of IL3 to activate G proteins in the dark and to be inactivated by light is common among peropsins of other protostomes and deuterostomes.

**Figure 4:**
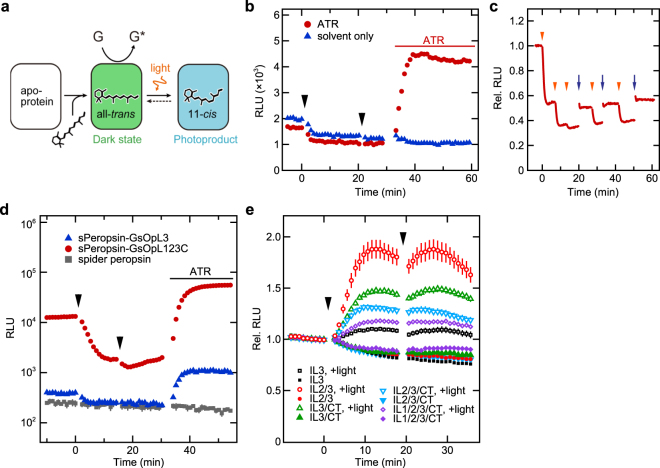
Peropsin mutant proteins serving as dark-active, light-inactivated GPCRs. (**a**) A schematic model for G protein activation by peropsin mutants. Apo-protein binds to all-*trans*-retinal and forms the dark state that activates G proteins. The dark state photo-converts to the photoproduct, or 11-*cis*-retinal-binding form, which does not efficiently activate G proteins. (**b**) GloSensor cAMP assay with sPeropsin-GsOpL3-expressing cells with addition of all-*trans*-retinal (ATR). All-*trans*-retinal (10 μM) or only solvent (ethanol) was added. (**c**) GloSensor cAMP assay with sPeropsin-GsOpL3-expressing cells preincubated with all-*trans*-retinal. Cells were illuminated with amber (arrowheads) and blue (arrows) light. (**d**,**e**) GloSensor cAMP assay with cells expressing different peropsin mutants that activate Gs (**d**) and Gi (**e**). ATR, all-*trans*-retinal (10 μM). Basal cAMP level was elevated by adding forskolin (1 μM) before the measurements (**e**). Averaged values for three samples are shown with error bars (s.e.m.) (**b**,**d**,**e)**. Cells were illuminated with amber light (arrowheads; **b**,**d**,**e**).

Figure 4a illustrates a schematic model for G protein activation by peropsin mutants based on our findings and those of our previous studies showing that the dark states and photoproducts of spider and amphioxus peropsins contain all-*trans* and 11-*cis* forms of chromophore, respectively^11,12^. Consistent with this model, cAMP levels in sPeropsin-GsOpL3-expressing cells without supplemental retinal exhibited a small but clear decrease upon illumination and an increase after addition of all-*trans*-retinal (Fig. 4b). The small decrease upon illumination can be explained by a photoreaction of a small amount of the peropsin mutant bound to endogenous retinal in standard medium containing bovine serum, according to our previous reports^18,19^. In addition, we observed that after the decrease of cAMP by illumination with orange light, blue light illumination caused an increase in cAMP level in sPeropsin-GsOpL3-expressing cells (Fig. 4c). This increase by blue light illumination would be due to photoconversion of the photoproduct into the dark state as we previously reported with spider peropsin^12^. These results are consistent with a model in which the dark state, or the all-*trans*-retinal-binding form, activates G proteins (Fig. 4a).

We next investigated whether additional replacement of other intracellular regions results in a larger amount of change in cAMP level. Regarding Gs-coupled mutants, the spider peropsin mutant containing all the intracellular regions, i.e. the first (IL1), second (IL2), and third intracellular loops and the C-terminal region (CT), of the jellyfish opsin (sPeropsin-GsOpL123C; Supplementary Fig. S3) showed higher cAMP levels in the dark, evoked a much larger light-dependent decrease in cAMP levels, and caused larger elevation of cAMP levels with the addition of all-*trans*-retinal, compared to sPeropsin-GsOpL3 (Fig. 4d). Rough estimation of the relative expression levels of peropsin mutants showed that the level of sPeropsin-GsOpL123C was higher than that of sPeropsin-GsOpL3 (Supplementary Fig. S5), suggesting that the increase in expression level of sPeropsin-GsOpL123C might contribute to the larger amount of change. In the case of Gi-coupled mutants, replacement of all intracellular regions (IL1/2/3/CT) by those of the C-terminal truncated MosOpn3^18^ exhibited a similar level of light-dependent increase in cAMP, compared to sPeropsin-GiOpL3 (Fig. 4e). We generated a set of mutants with different combinations of intracellular regions as shown in Fig. 4e and found that the mutant with MosOpn3 IL2 and IL3 (sPeropsin-GiOpL23; Supplementary Fig. S3) evoked the largest photoresponses. The expression level of sPeropsin-GiOpL23 was lower than that of sPeropsin-GiOpL3, suggesting that MosOpn3 IL2 might enhance G protein activation. Collectively, we found that replacement of intracellular regions in addition to IL3 resulted in a larger amount of change in cAMP level.

## Discussion

In this study, we examined two possible functions of peropsin, i.e. as a photoisomerase and light-sensing GPCR. First, the catalytic efficiency for retinal photoisomerisation was compared between spider peropsin and squid retinochrome, revealing that spider peropsin was much less efficient in our experiment (Fig. 1). Second, luminescence-based assays for intracellular Ca^2+^ and cAMP levels were performed but failed to show any considerable changes upon light stimulation in HEK293 cells expressing spider peropsin, suggesting that Gq, Gs, or Gi were not coupled to spider peropsin (Fig. 2). Interestingly, however, spider peropsin mutants in which the intracellular region(s) was replaced with that of a Gs-coupled opsin evoked a light-dependent decrease and an all-*trans*-retinal-dependent increase in cAMP levels (Figs 2, 4), indicating that the mutants could drive Gs signalling in the dark by binding to all-*trans*-retinal and were inactivated by absorption of light (see Fig. 4a). Similarly, the peropsin mutants with intracellular region(s) of a Gi-coupled opsin were suggested to be active and drive Gi signalling in the dark (Figs 2, 4). Such G-protein activation in the dark was also observed with amphioxus peropsin mutants with replacement of IL3 (Fig. 3). These findings suggest that the dark states (i.e. all-*trans*-retinal-binding forms) of these peropsin mutants activate G proteins and that their photoproducts (i.e. 11-*cis*-retnal-binding forms) are less efficient in G protein activation. Taken together, our findings suggest that wild-type peropsins might act as dark-active and light-inactivated GPCRs as discussed below.

The current study demonstrated that peropsin mutants containing the IL3 of other opsins can activate G proteins when they bind to all-*trans*-retinal as a chromophore (Figs 3, 4), providing insight into the conformational characteristics of wild-type peropsins. The scheme for bovine rhodopsin to convert into the active form, or Meta II state, is that isomerisation of the chromophore into all-*trans* form causes rearrangement of transmembrane α-helices, which in turn leads to conformational changes in intracellular regions bound to specific G proteins^20–22^. Thus, the specific arrangement of the transmembrane helices in the Meta II state is essential to activate G proteins. Our results suggest that the all-*trans*-retinal-binding forms of the peropsin mutants, which activate G proteins, would exhibit an arrangement of the transmembrane helices similar to the Meta II state, leading to conformation of the introduced intracellular loop(s) to activate G proteins. In addition, because the wild-type spider and amphioxus peropsins have the same amino acid sequences as their mutants in the transmembrane regions, the all-*trans*-retinal-binding forms of the wild-type peropsins may also have an arrangement of the transmembrane helices that is similar to the all-*trans*-binding active form (i.e. Meta II state) of bovine rhodopsin. Further studies are needed to evaluate the affinities of intracellular regions of these peropsins to G proteins other than Gs, Gi, and Gq. Recently, it was reported that human peropsin in keratinocytes is involved in increasing intracellular Ca^2+^ upon illumination with violet light^23^. It will be of interest to determine whether human peropsin itself activates G proteins in keratinocytes.

The unique property of peropsin mutants as an all-*trans*-retinal-dependently dark-active and light-inactivated GPCR shows their potential as a novel type of optogenetic tool. Because HEK293 cells expressing peropsin mutants exhibited light-dependent changes in cAMP levels in serum-containing medium with no supplementation of retinal (Fig. 4b,d,e), the peropsin mutants may spontaneously activate G proteins, without light stimulation, in the presence of all-*trans*-retinal at an endogenous concentration in mammalian bodies. Peropsin mutants evoked light-dependent changes in cAMP levels in a sustained manner, although slow recovery of cAMP levels was observed in most cases (Figs 2–4). Such slow recovery might be partly due to conversion of the photoproduct to the dark state by replacement of the chromophore as observed for wild-type spider peropsin (Fig. 1c) and/or an intrinsic mechanism(s) involved in recovery of cAMP level in HEK293 cells. Further studies will be needed to understand how the peropsin mutants act *in vivo* in detail. Various types of opsins have been proposed to be useful as dark-inactive, light-activated GPCRs for optogenetic manipulation of G protein signalling cascades^24–26^. To our knowledge, the peropsin mutants are the first optogenetic tools demonstrated to drive G protein signalling cascades spontaneously in the dark and be inactivated by light in living cells.

## Methods

### Construction of expression plasmid

Peropsin mutants were designed based on previous studies^17,24,27^ and generated by amplifying DNA fragments that overlap each other by 15–20 base pairs and fused by polymerase chain reaction. Fused fragments were tagged with the rho 1D4 epitope sequence (ETSQVAPA) and inserted into the multiple cloning site of pcDNA3.1 plasmid vector (Invitrogen, Carlsbad, CA, USA). The expression constructs of squid retinochrome^28^, spider^12^ and amphioxus^11^ peropsins, human Opn4^14^, box jellyfish opsin^15^, and C-terminal truncated MosOpn3^18^, each possessing the C-terminal 1D4 epitope sequence, were also used.

### Spectroscopic analysis of retinal isomerisation

Expression of opsins was performed as previously described^16,29^. Briefly, HEK293S cells were transfected with expression constructs by the calcium phosphate method and incubated for 2 days in 5% CO_2_ incubator at 37 °C. Opsin-containing cell membranes were collected by centrifugation and opsin-based pigments were constituted by overnight incubation with excess all-*trans*-retinal at 4 °C. The pigments were extracted with 1% (w/v) dodecyl β-D-maltoside (DM) in 50 mM HEPES buffer (pH 6.5) containing 140 mM NaCl (buffer A), bound to 1D4-agarose, washed using buffer A with 0.02% DM for retinochrome or with 0.1% DM and 0.1 mg/mL L-α-phosphatidylcholine from egg yolk^19,30^ for peropsin, and eluted with wash buffers containing the peptide corresponding to the 1D4 epitope sequence. Absorption spectra of the purified samples were measured using a spectrophotometer (UV2450, Shimadzu, Kyoto, Japan) at least three times and the averaged spectra were calculated. A 1-kW halogen lamp was used for illumination of samples with Y-52 or O-56 glass cutoff filters (AGC TECHNO GLASS, Shizuoka, Japan). The concentrations of retinal and squid retinochrome were determined using the extinction coefficients of 17,900 (water solution) and 60,800 M^−1^ cm^−1^, respectively^31,32^. The concentration of spider peropsin was tentatively estimated under the assumption that its extinction coefficient is similar to that of squid retinochrome.

### Bioluminescent reporter assays for Ca^2+^ and cAMP

A luminescent Ca^2+^ assay was conducted as described previously^14^ with minor modifications. Briefly, HEK293 cells were seeded into 96-well plates (25,000 cells per well) in DMEM containing 10% fetal bovine serum (FBS). After overnight incubation, cells were transfected with opsin constructs and the expression plasmid containing a DNA sequence coding the mitochondrially targeted aequorin^14^ using Lipofectamine 2000 (Invitrogen) according to the manufacturers’ instructions. On the following day, the medium was replaced with L-15 medium without phenol red (Invitrogen) containing 10% FBS, 10 μM coelenterazine *h* (Biotium), and 10 µM all-*trans*-retinal (peropsin) or 9-*cis* retinal (Opn4). Following 2-h incubation, luminescence was measured using a plate reader (FLUOstar Optima, BMG Labtech, Ortenburg, Germany). The cells were stimulated with white light from a Xe ramp for 2 s.

A GloSensor cAMP assay with 35-mm dishes (Figs 2b,c, 3, 4c) was carried out as described previously^16^. The samples were stimulated with a green (510 nm) light-emitting diode (LED) light for 5 s (Figs 2, 3) or amber (594 nm) and blue (450 nm) LED lights for 5 s (Fig. 4c). For the 96-well-based assay (Fig. 4b,d,e), HEK293S cells were seeded into a 96-well plate (20,000 cells per well), incubated overnight, and transfected with 50 ng of an opsin construct and 50 ng of GloSensor 22 F plasmid (Promega, Madison, WI, USA) per well by using polyethylenimine. After overnight incubation, the medium was replaced with a CO_2_-independent DMEM containing 10% FBS and 2% GloSensor cAMP Reagent (Promega). Luminescence was measured using a plate reader (FLUOstar Omega, BMG Labtech). The cells were stimulated with an amber (594 nm) LED light for 1 min.

## Electronic supplementary material

Supplementary Information

## References

[1] Terakita A (2005). The opsins. Genome Biol.

[2] Terakita A, Nagata T (2014). Functional properties of opsins and their contribution to light-sensing physiology. Zoolog Sci.

[3] Hao W, Fong HK (1999). The endogenous chromophore of retinal G protein-coupled receptor opsin from the pigment epithelium. J Biol Chem.

[4] Hara T, Hara R (1968). Regeneration of squid retinochrome. Nature.

[5] Hara T, Hara R (1967). Rhodopsin and retinochrome in the squid retina. Nature.

[6] Terakita A, Hara R, Hara T (1989). Retinal-binding protein as a shuttle for retinal in the rhodopsin-retinochrome system of the squid visual cells. Vision Res.

[7] Chen P (2001). A photic visual cycle of rhodopsin regeneration is dependent on Rgr. Nat Genet.

[8] Radu RA (2008). Retinal pigment epithelium-retinal G protein receptor-opsin mediates light-dependent translocation of all-trans-retinyl esters for synthesis of visual chromophore in retinal pigment epithelial cells. J Biol Chem.

[9] Sun H, Gilbert DJ, Copeland NG, Jenkins NA, Nathans J (1997). Peropsin, a novel visual pigment-like protein located in the apical microvilli of the retinal pigment epithelium. Proc Natl Acad Sci USA.

[10] Davies WIL (2015). An extended family of novel vertebrate photopigments is widely expressed and displays a diversity of function. Genome Research.

[11] Koyanagi M, Terakita A, Kubokawa K, Shichida Y (2002). Amphioxus homologs of Go-coupled rhodopsin and peropsin having 11-cis- and all-trans-retinals as their chromophores. FEBS Lett.

[12] Nagata T, Koyanagi M, Tsukamoto H, Terakita A (2010). Identification and characterization of a protostome homologue of peropsin from a jumping spider. J Comp Physiol A Neuroethol Sens Neural Behav Physiol.

[13] Hara T, Hara R (1973). Isomerization of retinal catalysed by retinochrome in the light. Nat New Biol.

[14] Bailes HJ, Lucas RJ (2013). Human melanopsin forms a pigment maximally sensitive to blue light (lambdamax approximately 479 nm) supporting activation of G(q/11) and G(i/o) signalling cascades. Proc Biol Sci.

[15] Koyanagi M (2008). Jellyfish vision starts with cAMP signaling mediated by opsin-G(s) cascade. Proc Natl Acad Sci USA.

[16] Sugihara T, Nagata T, Mason B, Koyanagi M, Terakita A (2016). Absorption Characteristics of Vertebrate Non-Visual Opsin, Opn3. PLoS One.

[17] Kim J-M (2005). Light-Driven Activation of β2-Adrenergic Receptor Signaling by a Chimeric Rhodopsin Containing the β2-Adrenergic Receptor Cytoplasmic Loops. Biochemistry.

[18] Koyanagi M, Takada E, Nagata T, Tsukamoto H, Terakita A (2013). Homologs of vertebrate Opn3 potentially serve as a light sensor in nonphotoreceptive tissue. Proc Natl Acad Sci USA.

[19] Tsukamoto H, Terakita A, Shichida Y (2005). A rhodopsin exhibiting binding ability to agonist all-trans-retinal. Proc Natl Acad Sci USA.

[20] Choe, H.-W. *et al*. Crystal structure of metarhodopsin II. *Nature* **471**, 651–655, doi:10.1038-nature09789 (2011). 10.1038/nature09789 21389988

[21] Deupi X (2014). Relevance of rhodopsin studies for GPCR activation. Biochim Biophys Acta.

[22] Farrens DL, Altenbach C, Yang K, Hubbell WL, Khorana HG (1996). Requirement of Rigid-Body Motion of Transmembrane Helices for Light Activation of Rhodopsin. Science.

[23] Toh PPC, Bigliardi-Qi M, Yap AMY, Sriram G, Bigliardi P (2016). Expression of peropsin in human skin is related to phototransduction of violet light in keratinocytes. Experimental Dermatology.

[24] Airan RD, Thompson KR, Fenno LE, Bernstein H, Deisseroth K (2009). Temporally precise *in vivo* control of intracellular signalling. Nature.

[25] Koyanagi M, Terakita A (2014). Diversity of animal opsin-based pigments and their optogenetic potential. Biochim Biophys Acta.

[26] Spangler SM, Bruchas MR (2017). Optogenetic approaches for dissecting neuromodulation and GPCR signaling in neural circuits. Current Opinion in Pharmacology.

[27] Yamashita T, Terakita A, Shichida Y (2000). Distinct roles of the second and third cytoplasmic loops of bovine rhodopsin in G protein activation. J Biol Chem.

[28] Terakita A, Yamashita T, Shichida Y (2000). Highly conserved glutamic acid in the extracellular IV-V loop in rhodopsins acts as the counterion in retinochrome, a member of the rhodopsin family. Proc Natl Acad Sci USA.

[29] Nagata T (2012). Depth perception from image defocus in a jumping spider. Science.

[30] Koyanagi M (2004). Bistable UV pigment in the lamprey pineal. Proc Natl Acad Sci USA.

[31] Szuts EZ, Harosi FI (1991). Solubility of retinoids in water. Arch Biochem Biophys.

[32] Hara T, Hara R (1982). Cephalopod retinochrome. Methods Enzymol.

