# Supplementary material for: An all-trans-retinal-binding opsin peropsin as a potential dark-active and light-inactivated G protein-coupled receptor
Source: Sci Rep. 2018 Feb 23;8:3535. doi: 10.1038/s41598-018-21946-1 (PMC5824942; doi:10.1038/s41598-018-21946-1)
Supplement: Supplementary file 1 — Supplementary Information [file 41598_2018_21946_MOESM1_ESM.pdf]

Supplementary Information for

An all-*trans*-retinal-binding opsin peropsin as a potential dark-active and light-inactivated G protein-coupled receptor

Takashi Nagata[a], Mitsumasa Koyanagi[a,b], Robert Lucas[c], Akihisa Terakita[a,b,]

[a]Department of Biology and Geosciences, Graduate School of Science, Osaka City University, 3-3-138 Sugimoto, Sumiyoshi-ku, Osaka 558-8585, Japan

[b]The OCU Advanced Research Institute for Natural Science and Technology (OCARINA), Osaka City University, 3-3-138 Sugimoto, Sumiyoshi-ku, Osaka 558-8585, Japan.

[c]Faculty of Biology, Medicine and Health, The University of Manchester, Manchester M13 9PT, UK

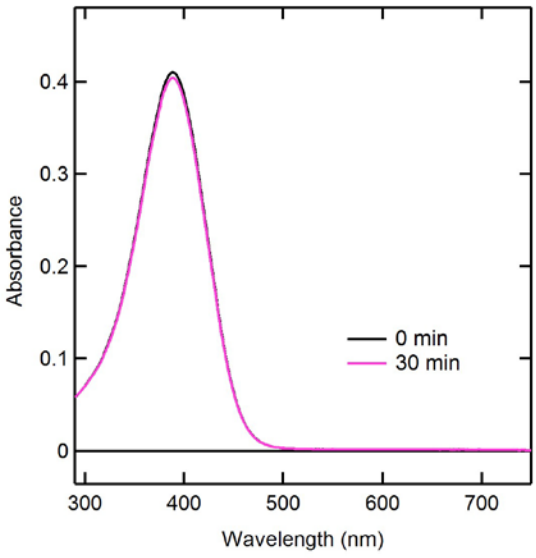

Supplementary figure S1: Absorption spectra of all-*trans*-retinal with no opsin before and after illumination with yellow light.

Spectroscopic analysis was performed in the same condition as Fig. 1a but without retinochrome.

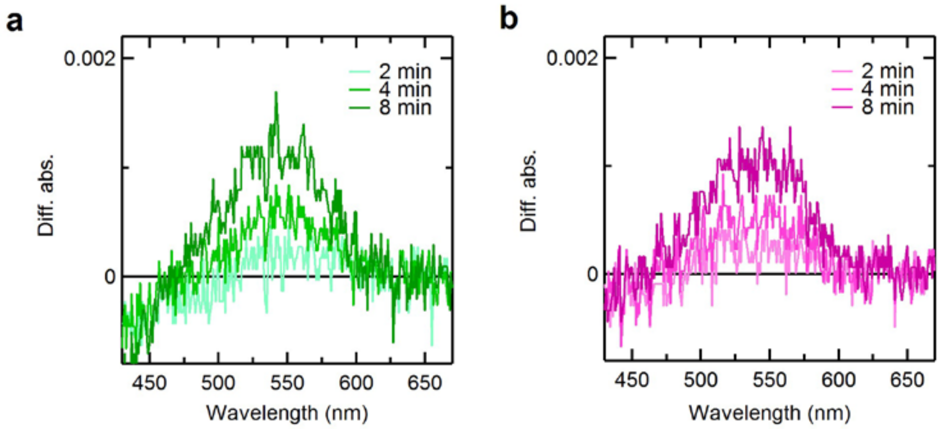

Supplementary figure S2: The recovery process of the dark state of spider peropsin.

Difference spectra of the same sample as in Fig. 1b showing the recovery process of the dark state of spider peropsin (λmax ≈540 nm) after the illumination for 3 (**a**) and 30 minutes (**b**). Absorption spectra were measured 1 minute after illumination (spectrum 1) and after incubation in the dark for 2, 4, and 8 minutes (spectra 2-4, respectively), and then the difference spectra were calculated by subtracting spectrum 1 from spectra 2-4.

a

```
                                                                 IL1
spider peropsin          MDDNMSEIALADDMSTLSTQEPSENVYPYVFPLSTHTIVGTYLIIIGILGTLGNGLVLVT 60
sPeropsin-GsOpL3         MDDNMSEIALADDMSTLSTQEPSENVYPYVFPLSTHTIVGTYLIIIGILGTLGNGLVLVT 60
sPeropsin-GsOpL123C      MDDNMSEIALADDMSTLSTQEPSENVYPYVFPLSTHTIVGTYLIIIGILGTLGNGLVLVT 60
sPeropsin-β2ARL3         MDDNMSEIALADDMSTLSTQEPSENVYPYVFPLSTHTIVGTYLIIIGILGTLGNGLVLVT 60
sPeropsin-GiOpL3         MDDNMSEIALADDMSTLSTQEPSENVYPYVFPLSTHTIVGTYLIIIGILGTLGNGLVLVT 60
sPeropsin-GiOpL23        MDDNMSEIALADDMSTLSTQEPSENVYPYVFPLSTHTIVGTYLIIIGILGTLGNGLVLVT 60
sPeropsin-GiOpL123C      MDDNMSEIALADDMSTLSTQEPSENVYPYVFPLSTHTIVGTYLIIIGILGTLGNGLVLVT 60

                             IL1
spider peropsin          FLRFRVLVTPTTLLLVNLAVSDLGLILFGFPFSASSSLSAKWIFGEGGCQWYAFMGFLFG 120
sPeropsin-GsOpL3         FLRFRVLVTPTTLLLVNLAVSDLGLILFGFPFSASSSLSAKWIFGEGGCQWYAFMGFLFG 120
sPeropsin-GsOpL123C      FYRLRHKLAFTTLLLVNLAVSDLGLILFGFPFSASSSLSAKWIFGEGGCQWYAFMGFLFG 120
sPeropsin-β2ARL3         FLRFRVLVTPTTLLLVNLAVSDLGLILFGFPFSASSSLSAKWIFGEGGCQWYAFMGFLFG 120
sPeropsin-GiOpL3         FLRFRVLVTPTTLLLVNLAVSDLGLILFGFPFSASSSLSAKWIFGEGGCQWYAFMGFLFG 120
sPeropsin-GiOpL23        FLRFRVLVTPTTLLLVNLAVSDLGLILFGFPFSASSSLSAKWIFGEGGCQWYAFMGFLFG 120
sPeropsin-GiOpL123C      MSKDMQLWTPTTLLLVNLAVSDLGLILFGFPFSASSSLSAKWIFGEGGCQWYAFMGFLFG 120

                                            IL2
spider peropsin          SAHIGTLALLALDRYLIACRISLRGKLTFKR--YTQMITVVWTYAFFWALMPLLGWGRYG 178
sPeropsin-GsOpL3         SAHIGTLALLALDRYLIACRISLRGKLTFKR--YTQMITVVWTYAFFWALMPLLGWGRYG 178
sPeropsin-GsOpL123C      SAHIGTLALLALDRYFTVCRPFVATAIHGSMRNYTQMITVVWTYAFFWALMPLLGWGRYG 180
sPeropsin-β2ARL3         SAHIGTLALLALDRYLIACRISLRGKLTFKR--YTQMITVVWTYAFFWALMPLLGWGRYG 178
sPeropsin-GiOpL3         SAHIGTLALLALDRYLIACRISLRGKLTFKR--YTQMITVVWTYAFFWALMPLLGWGRYG 178
sPeropsin-GiOpL23        SAHIGTLALLALERYCLISRPFSSRNLSRKG--YTQMITVVWTYAFFWALMPLLGWGRYG 178
sPeropsin-GiOpL123C      SAHIGTLALLALERYCLISRPFSSRNLSRKG--YTQMITVVWTYAFFWALMPLLGWGRYG 178

                                                                         IL3
spider peropsin          LEPSVTTCTIDWQHNDSSYKSFLIVYFVLGFMVPFAIIAVSYIAIARRVGKKSKERPVVR 238
sPeropsin-GsOpL3         LEPSVTTCTIDWQHNDSSYKSFLIVYFVLGFMVPFAIIAVSYILVQGEMKNMRGRAAQLF 238
sPeropsin-GsOpL123C      LEPSVTTCTIDWQHNDSSYKSFLIVYFVLGFMVPFAIIAVSYILVQGEMKNMRGRAAQLF 240
sPeropsin-β2ARL3         LEPSVTTCTIDWQHNDSSYKSFLIVYFVLGFMVPFAIIAVSYIRVFQEAKRQLQKIDKSE 238
sPeropsin-GiOpL3         LEPSVTTCTIDWQHNDSSYKSFLIVYFVLGFMVPFAIIAVSYINIIVYMRRNSARVGRIN 238
sPeropsin-GiOpL23        LEPSVTTCTIDWQHNDSSYKSFLIVYFVLGFMVPFAIIAVSYINIIVYMRRNSARVGRIN 238
sPeropsin-GiOpL123C      LEPSVTTCTIDWQHNDSSYKSFLIVYFVLGFMVPFAIIAVSYINIIVYMRRNSARVGRIN 238

                                        IL3
spider peropsin          DLWTNERSVT-------------------------LMAFILIVTFFVAWSPYAVLCLWTI 273
sPeropsin-GsOpL3         GSESEAALKNIKAEKRHT-----------------LMAFILIVTFFVAWSPYAVLCLWTI 281
sPeropsin-GsOpL123C      GSESEAALKNIKAEKRHT-----------------LMAFILIVTFFVAWSPYAVLCLWTI 283
sPeropsin-β2ARL3         GRFHVQNLSQVEQDGRTGHGLRRSSKFCLKEHKALLMAFILIVTFFVAWSPYAVLCLWTI 298
sPeropsin-GiOpL3         RAEQRVT----------------------------LMAFILIVTFFVAWSPYAVLCLWTI 270
sPeropsin-GiOpL23        RAEQRVT----------------------------LMAFILIVTFFVAWSPYAVLCLWTI 270
sPeropsin-GiOpL123C      RAEQRVT----------------------------LMAFILIVTFFVAWSPYAVLCLWTI 270

                                                                      CT
spider peropsin          FAEPNTVPPFLTLIPPLFAKSSTVVNPLIYFLSNPKLRTAILSTLSCCNEAPIQNIELPD 333
sPeropsin-GsOpL3         FAEPNTVPPFLTLIPPLFAKSSTVVNPLIYFLSNPKLRTAILSTLSCCNEAPIQNIELPD 341
sPeropsin-GsOpL123C      FAEPNTVPPFLTLIPPLFAKSSTVVNPLIYFLSNKQFRRAVLRGVCGRIVGGNAIAPSST 343
sPeropsin-β2ARL3         FAEPNTVPPFLTLIPPLFAKSSTVVNPLIYFLSNPKLRTAILSTLSCCNEAPIQNIELPD 358
sPeropsin-GiOpL3         FAEPNTVPPFLTLIPPLFAKSSTVVNPLIYFLSNPKLRTAILSTLSCCNEAPIQNIELPD 330
sPeropsin-GiOpL23        FAEPNTVPPFLTLIPPLFAKSSTVVNPLIYFLSNPKLRTAILSTLSCCNEAPIQNIELPD 330
sPeropsin-GiOpL123C      FAEPNTVPPFLTLIPPLFAKSSTVVNPLIYFLSNTQFRAAFTRVRNKGGVPTADQNTTTM 330

                              CT
spider peropsin          SPERAANNDAI      344
sPeropsin-GsOpL3         SPERAANNDAI      352
sPeropsin-GsOpL123C      AVEPGQTLASGTAES  358
sPeropsin-β2ARL3         SPERAANNDAI      369
sPeropsin-GiOpL3         SPERAANNDAI      341
sPeropsin-GiOpL23        SPERAANNDAI      341
sPeropsin-GiOpL123C      QRELTKSSRDMV     342
```

b

```
amphioxus peropsin       MDIPTETPYGAEEDIGESAGWRWTETDKNGFHKYDHLIVGLYLFVIGIIGTIENGITLAT 60
aPeropsin-GsOpL3         MDIPTETPYGAEEDIGESAGWRWTETDKNGFHKYDHLIVGLYLFVIGIIGTIENGITLAT 60
aPeropsin-GiOpL3         MDIPTETPYGAEEDIGESAGWRWTETDKNGFHKYDHLIVGLYLFVIGIIGTIENGITLAT 60

amphioxus peropsin       FSKFRSLRSPTTMLLVHLAIADLGICIFGYPFSGASSLRSHWLFGGVGCQWYGFNGMFFG 120
aPeropsin-GsOpL3         FSKFRSLRSPTTMLLVHLAIADLGICIFGYPFSGASSLRSHWLFGGVGCQWYGFNGMFFG 120
aPeropsin-GiOpL3         FSKFRSLRSPTTMLLVHLAIADLGICIFGYPFSGASSLRSHWLFGGVGCQWYGFNGMFFG 120

amphioxus peropsin       MANIGLLTCVAVDRYLVICRHDLVDKVNYNTYGVMAALGWLFAAFWAALPLVGWAEYALE 180
aPeropsin-GsOpL3         MANIGLLTCVAVDRYLVICRHDLVDKVNYNTYGVMAALGWLFAAFWAALPLVGWAEYALE 180
aPeropsin-GiOpL3         MANIGLLTCVAVDRYLVICRHDLVDKVNYNTYGVMAALGWLFAAFWAALPLVGWAEYALE 180

                                                                           IL3
amphioxus peropsin       PSGTACTINFQKNDSLYISYVTSCFVLGFVVPLAVMAFCYWQASCFVSKVLKGDIAGDLT 240
aPeropsin-GsOpL3         PSGTACTINFQKNDSLYISYVTSCFVLGFVVPLAVMAFCYWLVQGEMKNMRGRAAQLFGS 240
aPeropsin-GiOpL3         PSGTACTINFQKNDSLYISYVTSCFVLGFVVPLAVMAFCYWNIIVYMRRNSARVGRINRA 240

                              IL3
amphioxus peropsin       FPVAANVDWEYQNHFSKMCLAMVAAFVVAWTPYSVLFLFAAFWNPADIPAWLTLLPPLIA 300
aPeropsin-GsOpL3         ESEAALKNIKAEKRHTKMCLAMVAAFVVAWTPYSVLFLFAAFWNPADIPAWLTLLPPLIA 300
aPeropsin-GiOpL3         EQRVT-----------KMCLAMVAAFVVAWTPYSVLFLFAAFWNPADIPAWLTLLPPLIA 289

amphioxus peropsin       KSSALYNPIIYIIANRRFRNAICSMMKGQDPDVEDDEHADEHRVRSIEDNDKEIISMVNL 360
aPeropsin-GsOpL3         KSSALYNPIIYIIANRRFRNAICSMMKGQDPDVEDDEHADEHRVRSIEDNDKEIISMVNL 360
aPeropsin-GiOpL3         KSSALYNPIIYIIANRRFRNAICSMMKGQDPDVEDDEHADEHRVRSIEDNDKEIISMVNL 349

amphioxus peropsin       NMTV 364
aPeropsin-GsOpL3         NMTV 364
aPeropsin-GiOpL3         NMTV 353
```

Supplementary figure S3: Amino acid sequences of wild-type and mutant peropsins. (**a**) Spider peropsin and its mutants. (**b**) Amphioxus peropsin and its mutants. The first (IL1), second (IL2) and third (IL3) intracellular and C-terminal regions (CT) are indicated.

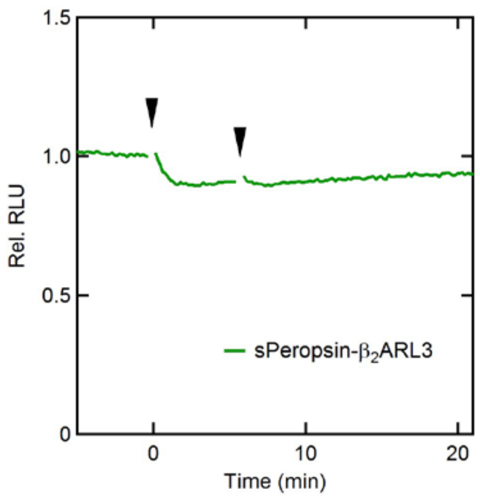

Supplementary figure S4: Light-dependent decrease in cAMP level in cells expressing sPeropsin-β_2ARL3.

The same data as in Fig. 2b is shown on a linear scale for clarity. Cells were illuminated with green light (arrowheads). The first illumination evoked an obvious decrease in cAMP level.

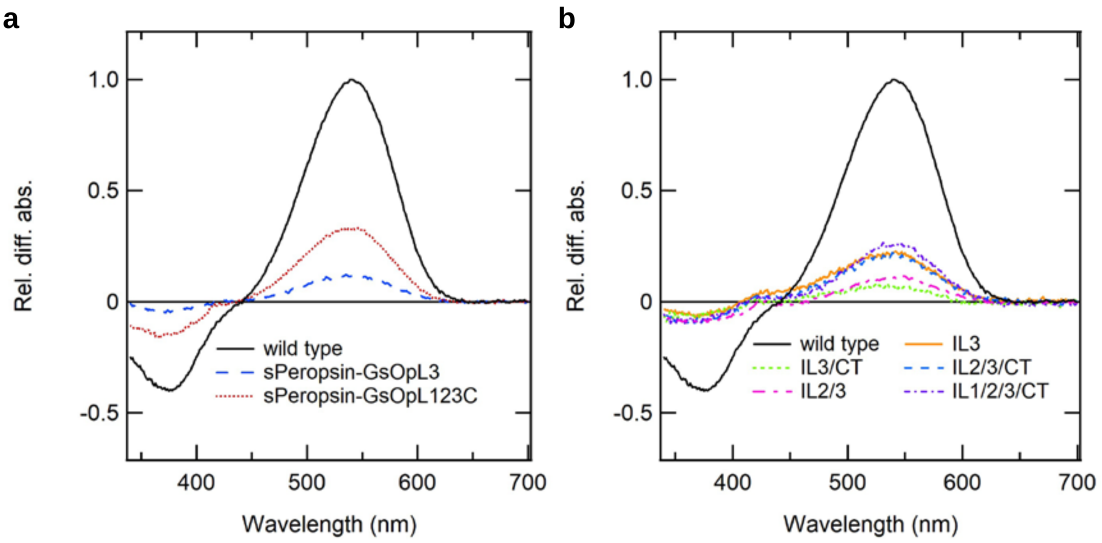

Supplementary figure S5: Difference spectra before-minus-after illumination showing expression levels of spider peropsin mutants in HEK293.

Difference spectra of Gs-coupled (**a**) and Gi-coupled (**b**) spider peropsin mutants relative to that of the wild type were obtained by subtracting absorption spectra after illumination from those before illumination. In the difference spectra, the positive peaks around 540 nm are mostly derived from the dark state present before illumination[1]. Expression levels of the mutants relative to that of wild type were estimated based on absorbance at 540 nm as follows: 11% (sPeropsin-GsOpL3), 33% (sPeropsin-GsOpL123C) for Gs-coupled mutants (**a**); 22% (IL3), 11% (IL2/3), 7% (IL3/CT), 21% (IL2/3/CT), 25% (IL1/2/3/CT) for Gi-coupled mutants (**b**). Cells expressing the opsins were prepared as for GloSensor cAMP assay. Cells in each 100-mm dish were transfected with 7.5 μg of an opsin plasmid and 7.5 μg of GloSensor plasmid using polyethylenimine. Pigments were constituted and extracted with dodecyl β-D-maltoside as described in the text. The extracted samples were diluted twice with buffer A and measured using the spectrophotometer. The O-56 glass cutoff filter was used for illuminating samples.

1 Nagata, T., Koyanagi, M., Tsukamoto, H. & Terakita, A. Identification and characterization of a protostome homologue of peropsin from a jumping spider. *J Comp Physiol A Neuroethol Sens Neural Behav Physiol* **196**, 51-59, doi:10.1007/s00359-009-0493-9 (2010).
